# Serum-soluble SDC1 in septic patients is rich in heparan sulfate, which affects the ELISA quantification values

**DOI:** 10.1016/j.bbrep.2025.102333

**Published:** 2025-10-30

**Authors:** Shogo Akahane, Riho Shimizu, Harue Suzuki, Hiroto Matsuura, Yoko Usami, Nau Ishimine, Takeshi Uehara, Kazuyoshi Yamauchi

**Affiliations:** a Department of Clinical Laboratory Investigation, Graduate School of Medicine, Shinshu University, Matsumoto, Japan; b Department of Laboratory Medicine, Shinshu University Hospital, Matsumoto, Japan; c Department of Biomedical Laboratory Sciences, School of Health Sciences, Shinshu University, Matsumoto, Japan

**Keywords:** Syndecan 1, Shedding, Inflammation, Matrix metalloproteinase, Thrombin, Heparinase

## Abstract

**Background:**

Soluble syndecan (sSDC) 1, which is released into the blood by shedding of the SDC1 ectodomain, is a potent biomarker of various inflammatory diseases and cancers. However, the characteristics of serum sSDC1 and methods for its quantification have not been thoroughly investigated. We investigated the properties of sSDC1 in the serum of patients with sepsis and their effects on measurement values.

**Methods:**

Serum sSDC1 levels in patients with and without sepsis were determined using dot blot analysis and enzyme-linked immunosorbent assay (ELISA). The molecular characteristics of serum sSDC1 were evaluated using western blotting and immunoprecipitation.

**Results:**

Serum sSDC1 ELISA values were more clearly able to distinguish between septic and non-septic patients than dot blot intensities. Serum sSDC1 mainly exists as the derivatives of the SDC1 ectodomain with molecular masses of 150–200 and 75 kDa, which were more prevalent in septic patients than non-septic patients. The former derivatives showed significant susceptibility to heparinase III. The presence of high-molecular-weight sSDC1 (>200 kDa), immunoprecipitated with an anti-heparan sulfate (HS) antibody, was also characteristic of the serum of patients with sepsis. The 150–200 and 75 kDa forms may have been derived from this high-molecular-weight HS-rich sSDC1. Importantly, the serum sSDC1 ELISA values for patients with sepsis showed a significant increase after treatment with heparinase III.

**Conclusions:**

sSDC1 molecules generated by sepsis-induced pathological shedding may be rich in HS and heterogeneous compared to those generated by physiological shedding. The HS content affected the ELISA quantification values of serum sSDC1.

## Highlights

• Serum sSDC1 ELISA values distinguish between septic and non-septic patients.
• Sepsis-induced pathologically shed serum sSDC1 is rich in heparan sulfate.
• The heparan sulfate moiety confers heterogeneity to serum sSDC1.
• The heparan sulfate content of serum sSDC1 affects the ELISA values.

## 1 Introduction

Syndecan (SDC)1, a type I transmembrane heparan sulfate proteoglycan, exists as a dimer on the cell surface and in the extracellular matrix of mammalian cells, especially epithelial cells [1,2]. The core protein of SDC1 consists of extracellular, transmembrane, and intracellular domains. The cytoplasmic domain is highly conserved, whereas the ectodomain is diverse, with the addition of heparan sulfate (HS)-glycosaminoglycan (GAG) and chondroitin sulfate (CS)-GAG chains via the O-glycosidic bond at the Ser residues of Ser-Gly pairs (Ser37, 45, and 48 for HS chains; Ser206 and 216 for CS chains) [3,4]. The monomeric core protein of SDC1 consists of 311 amino acids with a molecular weight (MW) of 33 kDa. However, the addition of GAG chains to the ectodomain enhances the molecular heterogeneity of the core proteins, accompanied by an increase in MW from 100 kDa to 300 kDa or higher [4].

SDC1 is responsible for pleiotropic biological functions, including the regulation of diverse cell behaviors (e.g., migration, adhesion, and invasion), signal transduction [[5], [6], [7], [8]], and lipoprotein metabolism, by cooperating as a co-receptor for other cell surface receptors, such as growth factor or lipoprotein receptors [9,10].

The ectodomain of SDC1 is known to be proteolytically shed from the cell surface and released into the bloodstream in a soluble form (sSDC1), even under physiological conditions to a small extent [[11], [12], [13]]. Although the detailed mechanisms of ectodomain shedding are not fully understood, several studies have provided evidence that SDC1 is a substrate for a variety of sheddases, including matrix metalloproteinases (MMPs), membrane-type MMPs, and a disintegrin and metalloproteinases (ADAM) family [[12], [13], [14], [15], [16]]. Serine proteases, such as thrombin and plasmin, have also been shown to be involved in the processing of the SDC1 ectodomain [13]. In addition, several specific cleavage sites have been identified or predicted in the SDC1 ectodomain of core proteins [[12], [13], [14]]. Endo et al. [15] demonstrated that human SDC1 can be cleaved at Gly245–Leu246 by MT1-MMP.

Shedding of the SDC1 ectodomain is accelerated in response to external stimuli and a wide range of pathological events, including inflammation, tumor progression, and wound healing, and it regulates these pathological processes [12,13,17]. Sanderson et al. [17,18] suggested that ectodomain shedding could be regulated by the HS content of the SDC1 core protein. Their view was that the loss of HS enhances the susceptibility of the core protein to proteolytic cleavage by MMPs, followed by the induction of core protein synthesis. However, the HS moiety is closely involved in signal transduction (e.g., inflammation) and has a significant effect on the physiological functions of SDC1 [13].

Pathologically enhanced shedding of the SDC1 ectodomain leads to elevated serum sSDC1 levels. Therefore, serum sSDC1 is expected to be a potent novel biomarker for various diseases. Evidence for the clinical usefulness of serum sSDC1 has been accumulating, and recent studies have emphasized that fluctuations in serum sSDC1 levels reflect the progression or remission of inflammatory diseases, most notably sepsis and diverse cancers [8,13,19]. Zhou et al. [19] suggested that serum sSDC1 levels may correlate with the severity of organ dysfunction in patients with sepsis and are a good predictor of the early diagnosis of sepsis.

As mentioned above, the ectodomain of SDC1 shows heterogeneity depending on the HS content and has multiple cleavage sites, strongly suggesting that a variety of SDC1 derivatives exist in serum. However, the molecular form of serum sSDC1 has not yet been studied in detail. Furthermore, the significance of serum sSDC1 as a biomarker can only be established if there is an appropriate method that can specifically measure disease-derived sSDC1; however, the reactivity and specificity of assays for heterogeneous serum sSDC1 molecules have yet to be investigated.

In this study, we characterized the patterns of sSDC1 molecules in the sera of septic and non-septic patients and assessed how these characteristic differences affected the measured values using a commercially available enzyme-linked immunosorbent assay (ELISA) kit.

## 2 Materials & methods

### 2.1 Materials

The rabbit anti-SDC1 polyclonal antibody (Ab) and horseradish peroxidase (HRP)-conjugated streptavidin were purchased from Proteintech (Rosemont, IL, USA). Mouse anti-SDC1 C-terminal vicinity recognition (C-term) and mouse anti-HS Abs were purchased from Sigma-Aldrich (St. Louis, MO, USA). HRP-conjugated anti-rabbit and anti-mouse IgG Abs were supplied by MBL Co. Ltd. (Nagoya, Japan). Heparinase III (Hep-III), O-glycosidase (O-Gly), and chondroitinase ABC (Ch-ABC) were purchased from Sigma-Aldrich.

### 2.2 Subjects

This study included 116 patients enrolled from April 2023 to March 2024 at Shinshu University Hospital (Matsumoto, Japan). Two hundred and sixty-five serum samples were obtained from 59 hospitalized patients with sepsis (68.3 ± 14.7 years), who were diagnosed according to the Sepsis-3 criteria [20] and a procalcitonin concentration >5.0 ng/mL. As a control, 57 serum samples were obtained from outpatients, who were verified to be without apparent disease (65.1 ± 14.7 years). All control patients showed normal results on the following serum laboratory tests: CRP concentration (<0.05 mg/dL); creatinine concentration (males, 0.65–1.07 mg/dL; females, 0.46–0.79 mg/dL); AST activity (13–30 U/L); ALT activity (males, 10–42 U/L; females, 7–23 U/L); LD activity (124–222 U/L); LDL-C concentration (65–120 mg/dL); HDL-C concentration (50–90 mg/dL); and TG concentration (40–150 mg/dL). All serum samples were aliquoted in small volumes and then stored at −80 °C until analysis. The present study was conducted in accordance with the principles of the Declaration of Helsinki and was approved by the Ethical Review Board of Shinshu University School of Medicine (approval number: 5762). Written informed consent was obtained from all the patients.

### 2.3 Determination of serum sSDC1 levels

Serum sSDC1 levels were determined using a commercial ELISA kit consisting of the B–B4 (DL-101) Ab as the capture Ab and a biotinylated B-D30 Ab as the detection Ab (Diaclone SAS, Besançon, France) [21,22].

### 2.4 Dot blotting (DB) analysis

Three microliters of each serum sample were blotted onto a polyvinylidene fluoride (PVDF) membrane using an immunoblotting apparatus. After fixing and blocking the membranes, the blots were incubated with rabbit anti-SDC1 polyclonal Ab, and then with HRP-conjugated anti-rabbit IgG. Dot intensities were quantified using Image J 1.45 software (National Institutes of Health, Bethesda, MD, USA).

### 2.5 Western blotting (WB) analysis

Serum samples were treated as described by Laemmli [23] and fractionated using 4–12 % gradient sodium dodecyl sulfate-polyacrylamide gel electrophoresis (SDS–PAGE) under non-reducing conditions. The separated proteins were electrophoretically transferred onto PVDF membranes. After blocking non-specific binding sites, the membranes were incubated with specific Abs against SDC1 as described above. Specific bands were detected using an enhanced chemiluminescence detection kit. Band intensities were quantified by densitometric analysis using ImageJ 1.45 software.

### 2.6 Immunoprecipitation (IP) analysis

IP analysis was performed using a direct method. Briefly, to prepare the immobilized Ab, 2.0 μg of anti-HS Ab was incubated with a 25-μL mixture of protein-G-coupled magnetic beads (Thermo Fisher Scientific) and 200 μL of phosphate-buffered saline (PBS) for 10 min at room temperature with constant rotation. Then, 200 μL of serum, diluted with PBS, was incubated with the immobilized Ab, followed by incubation for 2 h at room temperature with constant rotation. After collecting the supernatant and washing the beads, they were resuspended in SDS-PAGE sample buffer and boiled for 5 min, followed by WB analysis using an anti-SDC1 polyclonal Ab.

### 2.7 Statistical methods

Data are presented as the mean ± standard deviation. The relationships between each test value were evaluated using Spearman's rank correlation test. Differences between the two groups were assessed using the Wilcoxon signed-rank sum test or Mann-Whitney test. Statistical significance was set at *p < *0.05.

## 3 Results

### 3.1 Determination of serum sSDC1 levels

To detect pan-sSDC1, which is expected to be a heterogeneous molecule, in serum samples, we conducted DB analysis using an anti-SDC1 polyclonal Ab. The dot intensity increased proportionally with the sSDC1 concentration, with linearity up to approximately 400 ng/mL (Fig. 1). The within-run coefficients of variation (CVs) for the dot intensities of 50 and 100 ng/mL recombinant SDC1 in 10 replicates were 8.3 and 4.1 %, respectively. The between-run CV for 100 ng/mL recombinant SDC1 measured on 14 consecutive days was 7.3 %.

**Fig. 1:**
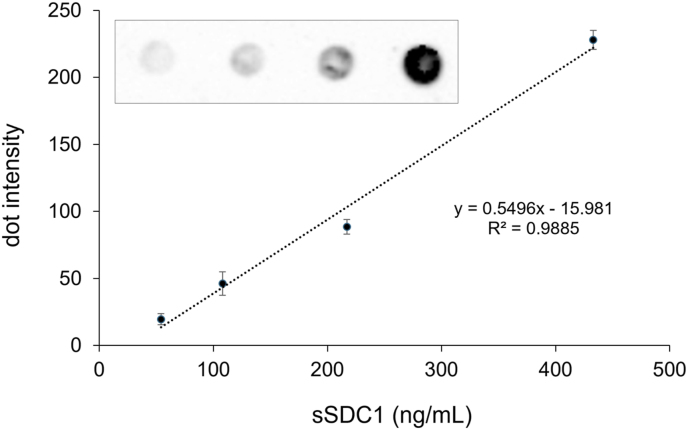
**Dilution linearity of the dot blot method for serum pan-SDC1.** Three microliters of serially diluted recombinant SDC1 (54.0, 108.0, 217.0, and 433.0 ng/mL) was blotted onto a polyvinylidene fluoride membrane, followed by incubation with an anti-SDC1 polyclonal Ab, as described in the Materials and Methods section. Dot intensities were quantified using Image J 1.45 software. The values shown are the means ± standard deviation (SD) from duplicate determinations in two independent experiments.

Dot intensities and ELISA values for serum sSDC1 were compared between patients with sepsis and controls. Immunoreactivity to an anti-SDC1 polyclonal Ab was also detected in the control group; however, no correlation was observed between the dot intensities and ELISA values (n = 36, *r*_*s*_ = 0.156; Fig. 2a). In sera from the septic group, the dot intensities were significantly correlated with the ELISA values (n = 69, *r*_*s*_ = 0.320, *p* = 0.0073; Fig. 2b). When limited to samples within the linear range of the present DB method, the correlation was more pronounced (n = 55, *r*_*s*_ = 0.424, *p* = 0.0013; Fig. 2c). However, although the dot intensities of the septic patient group were 1.3-fold higher than those of the control patient group (*p* = 0.0163) (Fig. 2d), the differences were not pronounced compared to those in the ELISA values between groups (4.5-fold higher, *p* = 2.5123 × 10^−10^) (Fig. 2e).

**Fig. 2:**
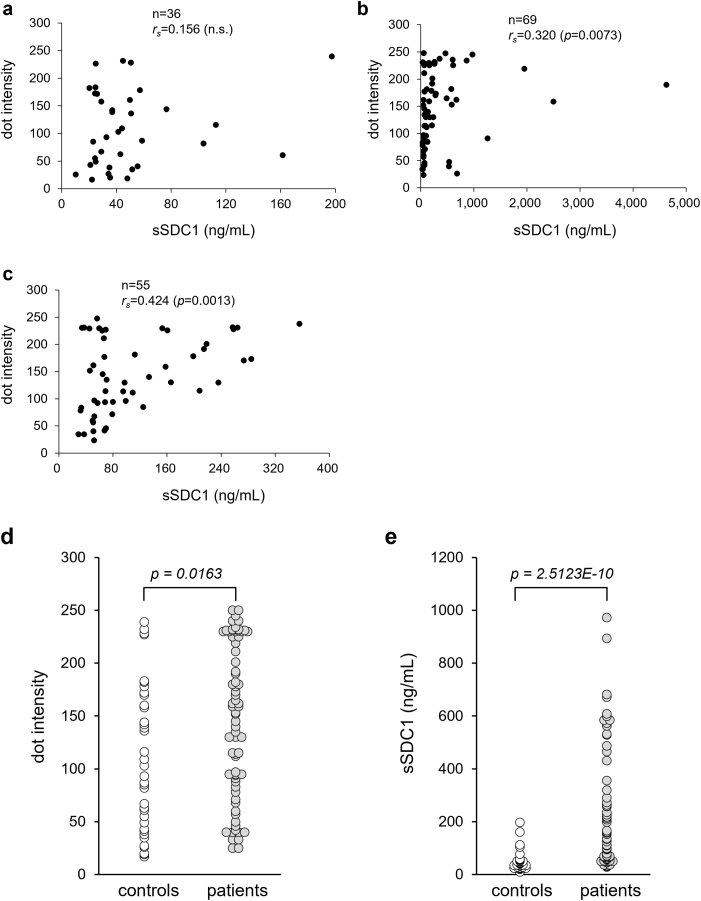
**Serum sSDC1 levels of septic patients and non-septic patients.** Correlation between enzyme-linked immunosorbent assay (ELISA) values of sSDC1 and dot intensities of pan-sSDC1 in sera from control patients (**a**), septic patients (**b**), or septic patients excluding the >400.0 ng/mL ELISA value (**c**). *r*_*s*_, Spearman's rank correlation coefficient. n.s., not significant. Comparison of dot intensities (**d**) and ELISA values (**e**) between control and septic patients.

### 3.2 WB analysis of serum sSDC1

To examine the differences in serum sSDC1 levels between control and septic patients, we performed WB analysis using various anti-SDC1 antibodies (Abs). The Abs used in the WB analysis are listed in Table 1.

**Table:** The epitope regions recognized by each antibody used in the WB analysis.

antibody (Ab)	host	clone	epitope region
polyclonal Ab	rabbit	N.A.	N.A.
ELISA capture Ab	mouse	B–B4	90-93 aa
ELISA detection Ab	mouse	B-D30	82-126 aa
C-terminal vicinity recognition Ab	mouse	587CT7.3.6.5	210-238 aa

N.A., not applicable.

Consistent with the results of the DB analysis, the sera of control patients showed significant immunoreactivity to the anti-SDC1 polyclonal Ab. Although the patterns for the control patients' sera were basically the same as those of the septic patients’ sera, visual inspection suggested higher intensities in the 150–200 and 75 kDa regions of the septic patients (Fig. 3a). Quantification confirmed that the 150–200 kDa region detected with the polyclonal Ab was 1.4-fold higher in septic patients than in controls (*p* = 0.0433), whereas no significant difference was observed for the 75 kDa region (Fig. 3b). The 150–200 kDa region also showed immunoreactivity to the ELISA capture Ab, ELISA detection Ab, and C-term Ab (Fig. 3, arrows), whereas the 75 kDa region showed significant immunoreactivity only with the ELISA detection Ab (Fig. 3, dotted arrows). Further characteristic differences between sera of control and septic patients were observed in the WB analysis using the ELISA detection Ab. First, the 150–200 kDa region in the sera of septic patients showed a broad pattern, whereas the corresponding region in control sera appeared as a sharp band of slightly lower MW (approximately 130 kDa) rather than as a broad band (Fig. 3, asterisk). Second, the immunoreactivities of the <150 kDa and >200 kDa regions in the sera of septic patients were significantly stronger than those in the sera of control patients.

**Fig. 3:**
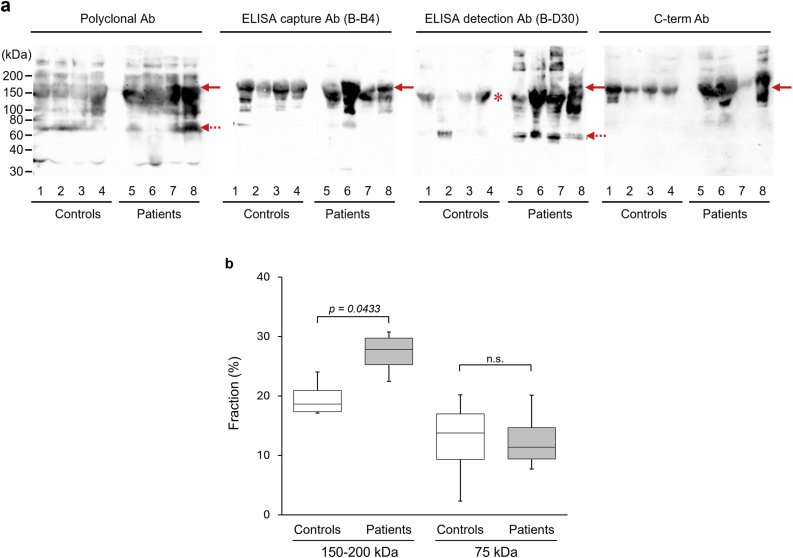
**Western blotting analysis of serum sSDC1** Four serum samples, each obtained from control patients (lanes 1, 2, 3, and 4) and septic patients (lanes 5, 6, 7, and 8), were separated by sodium dodecyl sulfate-polyacrylamide gel electrophoresis using a 4–12 % gradient gel under nonreducing conditions, followed by detection using an anti-SDC1 polyclonal Ab, ELISA capture Ab, ELISA detection Ab, or C-term Ab. The sera of patients with sepsis were diluted 4- or 10-fold in phosphate-buffered saline for western blotting analysis. The serum sSDC1 concentrations (ng/mL) for each sample were as follows: lane 1, 37.0; lane 2, 51.6; lane 3, 23.0; lane 4, 34.9; lane 5, 107.8; lane 6, 54.6; lane 7, 195.0; and lane 8, 121.7. Arrows, 150–200 kDa derivatives; dotted arrows, 75 kDa derivatives; asterisk, 130 kDa derivatives (**a**). Densitometric quantification of the polyclonal Ab signal was performed for the 150–200 kDa region (arrows) and the 75 kDa region (dotted arrows). Values represent the fraction (%) of each band relative to the sum of all sSDC1-reactive bands per lane. Box-and-whisker plots show data from four control (lanes 1–4) and four septic patients (lanes 5–8), with each value representing the mean of three independent analyses performed by different investigators (**b**).

### 3.3 Characteristics of the GAG chain of serum sSDC1 molecules

To compare the characteristics of the GAG chain of serum sSDC1 in control patients and septic patients, each pooled serum sample was incubated with Hep-III, O-Gly, or Ch-ABC and then subjected to WB analysis. We used pooled serum samples to minimize the influence of inter-individual variability on the molecular features of sSDC1.

Incubation with Hep-III significantly enhanced the intensities of the 150–200 and 75 kDa regions in the pooled septic serum, detected using an anti-SDC1 polyclonal Ab, in a manner dependent on Hep-III activity. Similar patterns were observed in the WB analysis of the pooled septic serum using other Abs (Fig. 4a). Enhancement of the 150–200 kDa region was also detected using the ELISA capture and C-term Abs (Fig. 4a, arrows), while enhancement of the 75 kDa region was also detected using the ELISA detection Ab (Fig. 4a, dotted arrows). These visual observations were confirmed by densitometric analyses, which demonstrated clear, dose-dependent increases in band intensities with increasing Hep-III concentrations (Fig. 4b–f).

**Fig. 4:**
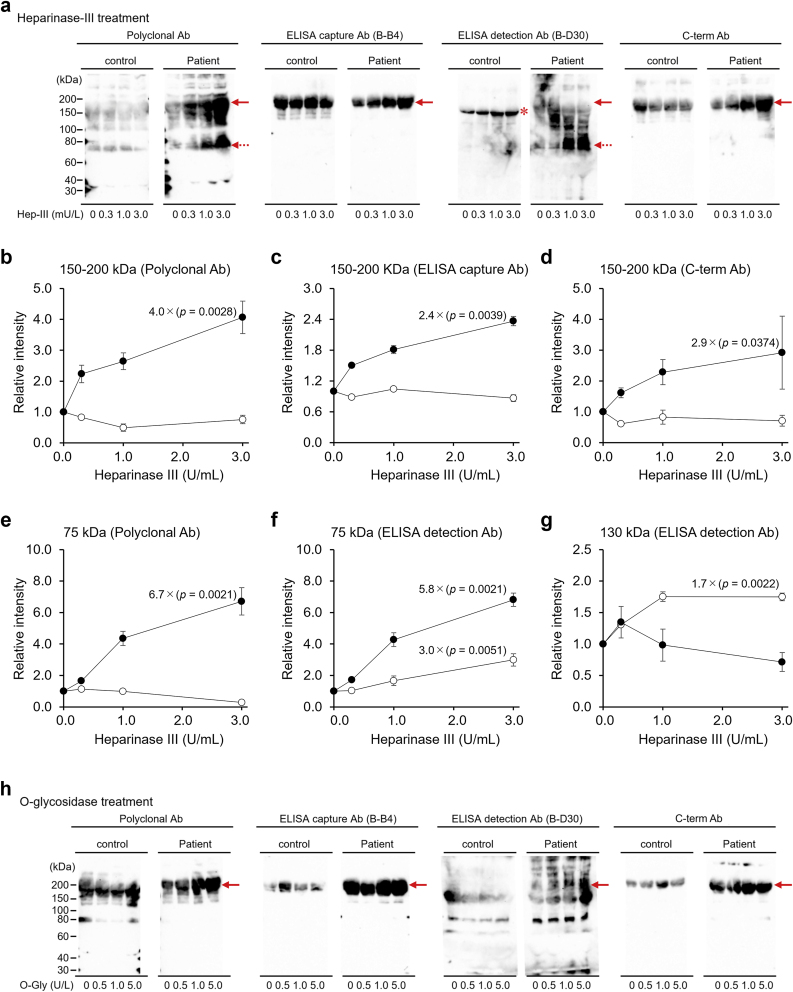

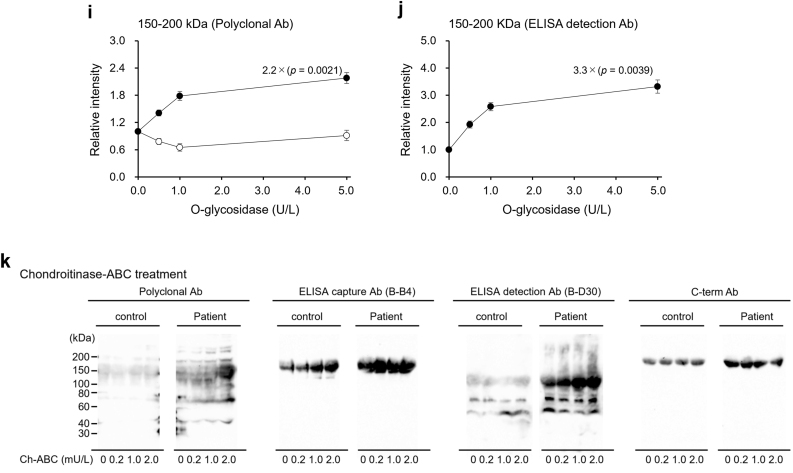
**Effect of enzyme treatment on serum sSDC1 molecules.** Pooled sera of control patients and septic patients, diluted with PBS to equalize the sSDC1 concentration, were incubated with Hep-III (0.3, 1.0, or 3.0 U/mL) (**a**), O-Gly (0.5, 1.0, or 5.0 U/L) (**h**), or Ch-ABC (0.2, 1.0, or 2.0 U/mL) (**k**) for 24 h at 37 °C. Similarly, sera were incubated with 3.0 U/mL heat-inactivated Hep-III, 5.0 U/L heat-inactivated O-Gly, or 2.0 U/mL heat-inactivated Ch-ABC as negative controls with zero enzyme activity. The mixtures were then subjected to WB analysis using an anti-SDC1 polyclonal Ab, ELISA capture Ab, ELISA detection Ab, or C-term Ab. Arrows, 150–200 kDa derivatives; dotted arrows, 75 kDa derivatives; asterisk, 130 kDa derivatives. The results of densitometric analyses after Hep-III treatment are presented for the 150–200 kDa region [using the anti-SDC1 polyclonal Ab (**b**), ELISA capture Ab (**c**), and C-term Ab (**d**)], for the 75 kDa region [using the anti-SDC1 polyclonal (**e**) and ELISA detection Abs (**f**)], and for the 130 kDa region [using the ELISA detection Ab (**g**)]. The results after O-Gly treatment are shown for the 150–200 kDa region [using the anti-SDC1 polyclonal Ab (**i**) and ELISA detection Ab (**j**)]. The corresponding band in the control serum was below the detection limit and thus not shown in panel (j). Closed circles indicate values for septic patients, whereas open circles indicate those for control patients. Each value represents the mean ± SD of three independent analyses performed by different investigators. Fold-induction values (relative to 0 U/mL or 0 U/L enzyme treatment) and corresponding p-values are indicated in each graph.

The intensities of the <150 and >200 kDa regions in the pooled septic serum detected using an anti-SDC1 polyclonal Ab were also enhanced by Hep-III treatment. Such Hep–III–dependent enhancements of the <150 kDa region, but not the >200 kDa region, were also detected using the ELISA detection Ab. Although the effect of Hep-III treatment on the pooled control serum was modest compared to the effect on the pooled septic serum, the enhancement of the 130 kDa region was detected using the ELISA detection Ab (Fig. 4a, asterisk). Densitometric analysis further revealed that, at higher Hep-III concentrations, the increase in signal intensity of this region was significantly greater in the pooled control serum than in the pooled septic serum (Fig. 4g).

Similar to the results of Hep-III treatment, O-Gly treatment significantly enhanced the intensity of the 150–200 kDa region in the pooled septic serum as O-Gly activity increased. This O-Gly-dependent enhancement was also detected using the ELISA detection Ab (Fig. 4h, arrows). In contrast, no significant change was observed in the intensity of the corresponding region detected with the ELISA capture Ab or the C-term Ab. Densitometric analyses further confirmed these observations, demonstrating dose-dependent increases in the 150–200 kDa region with increasing O-Gly concentrations (Fig. 4i and j).

Although incubation with Ch-ABC enhanced the reactivity of the anti-SDC1 polyclonal Ab to both pooled sera of control and septic patients and the reactivity of the detection Ab to the pooled septic serum, the effect was slight compared with that of Hep-III or O-Gly treatment (Fig. 4k).

### 3.4 Effect of the GAG chain of sSDC1 molecules on ELISA quantification

To further investigate the effects of the HS and CS chains of serum sSDC1 molecules on their measurement, we performed ELISA using the above-mentioned enzyme-treated pooled serum samples.

The sSDC1 levels in both pooled sera of control and septic patients increased in a manner dependent on Hep-III activity. This effect was more pronounced in pooled septic serum than in pooled control serum. Compared with their respective negative controls, the sSDC1 levels in the pooled sera of control patients and septic patients increased by 1.4-fold (*p* = 0.0009) and 2.5-fold (*p* = 1.3268 × 10^−10^), respectively, after treatment with 3.0 U/mL Hep-III (Fig. 5a). Incubation with O-Gly also increased serum sSDC1 levels. However, the degree of increase in sSDC1 levels was approximately 1.5-fold (*p* = 8.78317 × 10^−5^) that of the levels in the respective negative controls for both control and septic patients, and no significant difference was observed between these two groups (Fig. 5b). Ch-ABC incubation slightly but significantly increased the sSDC1 levels in the pooled septic serum (*p* = 0.0168), but had no effect on those in the pooled control serum (Fig. 5c).

**Fig. 5:**
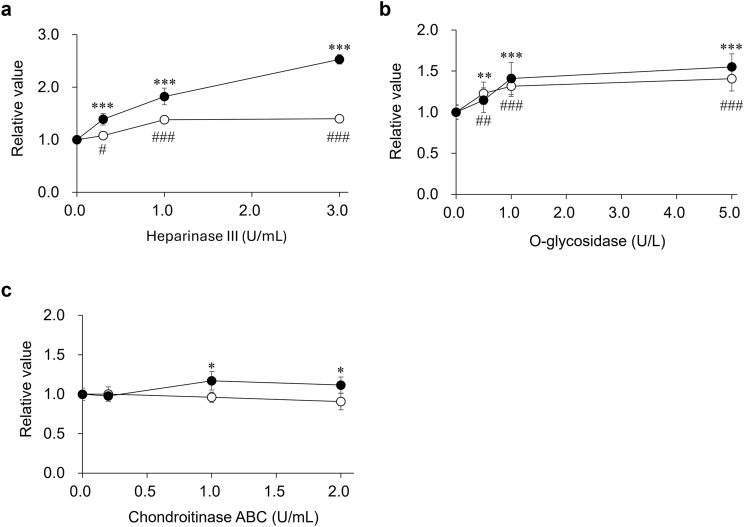
**Effect of enzyme treatment on the measurement of serum sSDC1.** Pooled sera of control patients and septic patients, diluted with PBS to equalize the sSDC1 concentration, were incubated with Hep-III (0.3, 1.0, or 3.0 U/mL) (**a**), O-Gly (0.5, 1.0, or 5.0 U/L) (**b**), or Ch-ABC (0.2, 1.0, or 2.0 U/mL) (**c**) for 24 h at 37 °C. Similarly, sera were incubated with 3.0 U/mL heat-inactivated Hep-III, 5.0 U/L heat-inactivated O-Gly, or 2.0 U/mL heat-inactivated Ch-ABC as negative controls with zero enzyme activity. The mixtures were then subjected to ELISA to detect sSDC1. Closed circles indicate values for septic patients, whereas open circles indicate those for control patients. The values (means ± SD) obtained from duplicate determinations, each in three independent experiments) are expressed relative to those of each negative control. ∗∗∗*p* < 0.001, ∗∗*p* < 0.01, and ∗*p* < 0.05 compared with the negative control for the pooled serum of septic patients. ^###^*p* < 0.001, ^##^*p* < 0.01, and ^#^*p* < 0.05, compared to the negative control for the pooled serum of control patients.

### 3.5 Characteristics of pathological sSDC1 molecules determined by IP analysis

To further characterize the pathologically shed sSDC1 molecules in the sera of patients with sepsis, we conducted IP analysis using an anti-HS Ab immobilized on protein G-coupled magnetic beads.

Several prominent bands with a high MW (>200 kDa) were detected using the anti-SDC1 Ab in the IP eluate of pooled septic serum. In contrast, these bands were barely observed in the IP eluate of pooled control serum (Fig. 6a). Quantitative densitometric analysis of the IP eluates confirmed that the band intensities were approximately 44-fold higher in pooled septic serum than in pooled control serum (*p* = 0.0022) (Fig. 6b). To validate the functionality and specificity of the anti-HS Ab, we performed a dot blot assay using serum samples with or without 3.0 U/mL Hep-III treatment. Strong signals were observed in untreated serum, whereas they were abolished after Hep-III treatment (Fig. 6c). Together, these results validate the performance of the Ab and support the interpretation that the weak control signals reflect genuinely low HS content.

**Fig. 6:**
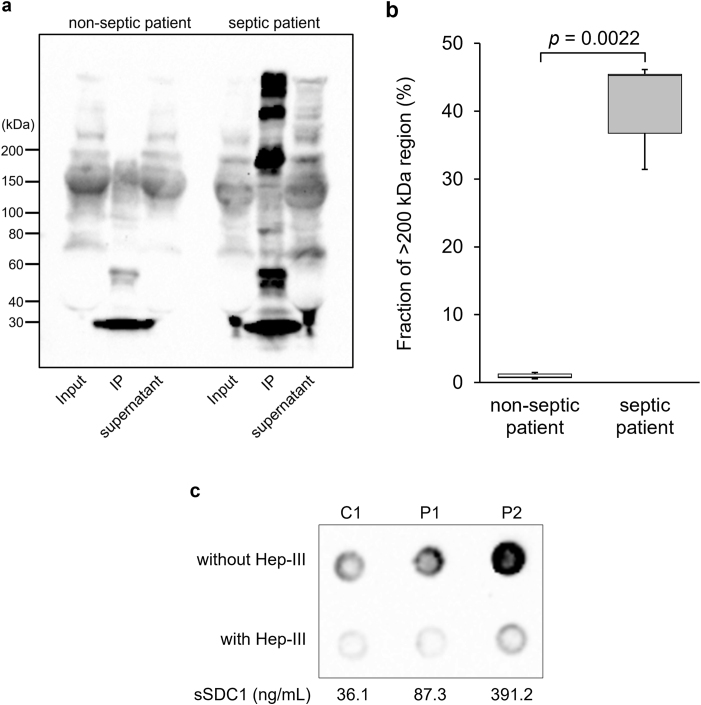
**Immunoprecipitation analysis** As described in the Materials and Methods section, pooled sera of control patients and septic patients, diluted with PBS to equalize the sSDC1 concentration, were incubated with an anti-HS Ab immobilized on protein-G-coupled magnetic beads. The precipitates and supernatants were then subjected to WB analysis using a polyclonal anti-SDC1 Ab (**a**). “Input” indicates serum mixed with an equal volume of PBS and used as the immobilized anti-HS Ab solution. Densitometric quantification of the >200 kDa region in the immunoprecipitated eluates (IP) is shown (**b**). Values represent the fraction (%) of the >200 kDa region relative to the sum of all sSDC1-reactive bands in each lane, excluding immunoglobulin heavy and light chain bands derived from the immunoprecipitation procedure. Box-and-whisker plots show data from pooled control and pooled septic sera, with each value representing the mean of three independent analyses performed by different investigators. A dot blot assay was also performed using serum samples from one control patient (C1) and two septic patients (P1, P2) with or without 3.0 U/mL Hep-III treatment to validate the functionality and specificity of the anti-HS Ab (**c**).

## 4 Discussion

Cumulative evidence has clearly demonstrated that the shedding of cell-surface SDC1 is prominently enhanced under pathological conditions [4,12,13,[17], [18], [19]]; however, the details of its underlying mechanisms and pathophysiological consequences remain unclear. Characterization of serum sSDC1 molecules, the products derived from shedding, may provide new insights into these issues.

Similar to the serum from septic patients, the serum from control patients showed significant dot intensity, providing evidence for the previous suggestion that substantial amounts of sSDC1 derivatives may be present even in the serum of healthy subjects [[11], [12], [13]]. Although DB and ELISA results correlated to some extent, the ELISA values were more useful for differentiating septic patients from non-septic patients. These findings implicitly suggest that pathologically shed SDC1 in the serum of septic patients may have different properties from physiologically shed SDC1. Indeed, several apparent differences in WB patterns using the ELISA Abs were observed between septic and control patients.

The 150–200 kDa region in the serum of septic patients was immunoreacted with all anti-SDC1 Abs used in the present study. The significant reactivity with the C-term Ab indicated that the corresponding derivatives may contain at least a portion of the region of amino acid residues 210–238 of the SDC1 ectodomain, an epitope of this Ab [24]. Thus, the 150–200 kDa derivatives may have a cleavage site downstream (C-terminal side) from the amino acid residue Asp210. Candidate sites for cleavage of the SDC1 ectodomain have been described in several studies [[12], [13], [14]]. Given the above requirements, among the candidate sites, Gly245, Asp236, and Arg230 may be plausible cleavage sites for the generation of 150–200 kDa derivatives. However, based on reactivity with the ELISA capture Ab, which recognizes the region of amino acid residues 90–93 of the SDC1 ectodomain [21], a cleavage site on the N-terminal side of this derivative was expected to be located upstream of amino acid residue 93. Ramani et al. [18] noted that the high-MW form of sSDC1 is difficult to transfer to a membrane or has less Ab accessibility than the low-MW form, thus creating the false impression that the amount of sSDC1 was increased by Hep-III treatment. Based on this idea, our results show that the 150–200 kDa region in the pooled septic serum was enhanced by Hep-III or O-Gly treatment and showed a broad band pattern compared to that in the pooled control serum. This may be attributed to the decrease in the molecular size of sSDC1 due to enzymatic degradation of its HS chains. Namely, the 150–200 kDa derivatives may be derived from HS-rich sSDC1 with a MW > 200 kDa. Such a reduction in molecular size could improve membrane transfer efficiency or Ab accessibility, resulting in the broadening of the 150–200 kDa region and enhancement of the <150 kDa region. We speculate that the <150 kDa derivatives may also be derived from HS-rich sSDC1 with a high-MW > 200 kDa or its derivatives (i.e., 150–200 kDa derivatives).

Previous studies proposed that the HS content of the SDC1 ectodomain regulates its shedding rate [17,18]. They showed that the presence of HS chains inhibits SDC1 shedding, whereas their loss or shortening promotes shedding by enhancing the susceptibility of the core protein to proteolytic cleavage by various enzymes, such as MMPs. They have also suggested that the pathological activation of heparanase, an endo-β-d-glycosidase, which is known to be upregulated in cancer, inflammation, and wound healing [25,26], may be responsible for the decreased HS content. Contrary to these previous findings, our results strongly indicated that pathologically shed SDC1 molecules, which are elevated in pooled septic serum, may be HS-rich and heterogeneous due to variations in HS content. In contrast, the finding that control serum showed a sharp 130 kDa band suggests that physiologically shed SDC1 may be a relatively homogeneous molecule with a nearly constant HS content. Our finding that the ELISA detection Ab could detect the enhancement of the <150 kDa region, including the 130 kDa derivative, but not the >200 kDa region, in the pooled control serum suggests that the access of this Ab to >200 kDa derivatives may be blocked by their abundant HS. This also supports the abovementioned hypothesis that serum sSDC1 in septic patients may be HS-rich. However, since the HS content of SDC1 may vary depending on the stage of sepsis, we plan to analyze time-related fluctuations in serum sSDC1 patterns of various patients in a subsequent study.

The 75 kDa region was reactive only with the ELISA detection Ab, suggesting that the corresponding derivatives may be part of the SDC1 ectodomain, which includes the region of amino acid residues 82–126 recognized by this Ab (information provided by Diaclone SAS). Based on our results, it is highly likely that these derivatives do not contain any binding sites for the HS or CS chains. In fact, although the 75 kDa region was enhanced by the Hep-III or O-Gly treatment, the derivative itself showed no sensitivity to these enzymes and no reactivity in the <75 kDa region, which may appear after enzymatic digestion. These findings also indicate that the 75 kDa derivative may be derived from HS-rich high-MW sSDC1. In addition, unlike the 150–200 kDa region, this low-MW region showed a narrow band, suggesting that this derivative may be comparatively homogeneous because it does not contain HS chains. Based on our results, company information about the ELISA detection Abs, and a previous review article [13], we hypothesize that Gly82 and Arg126 may be plausible candidates for cleavage sites of the N- and C-terminal domains of the SDC1 molecule, respectively. Gly82 is processed by MMP2, MMP9, and MMP14, whereas Arg126 is processed by thrombin. Although the reason why the 75 kDa derivative showed no reactivity with the ELISA capture Ab, which recognizes amino acid residues 90–93, is unclear, this derivative may not be detectable by ELISA. Further studies using mass spectrometry analysis are necessary to determine the exact cleavage sites on both the N- and C-terminal domains of the 150–200 and 75 kDa derivatives.

It is well known that sepsis disrupts the homeostasis between pro- and anti-coagulant pathways, resulting in systemic thrombin production, decreased anticoagulant activity, and suppression of fibrinolysis, a condition termed sepsis-induced coagulopathy [27]. If the 75 kDa derivative was derived from the cleavage of the SDC1 ectodomain by thrombin, our finding that serum from septic patients had stronger reactivity than control patient serum for the 75 kDa region may be attributed to the abnormal elevation of thrombin activity in septic patients. The presence of these derivatives even in control serum may be explained by the use of thrombin-containing blood collection tubes or by physiological activation of thrombin *in vivo*. Supporting this, Supplementary Fig. 1 shows that the 75 kDa region in sera from healthy volunteers collected in tubes without thrombin was enhanced upon the addition of thrombin, demonstrating that thrombin can directly increase the generation of this fragment in serum. In view of these aspects, we cannot rule out the possibility that the consumption of antithrombin III, which is caused by binding with sSDC1 [28] or by excessive thrombin activation in patients with sepsis, may more prominently manifest the effect of the thrombin added as an anticoagulant in the blood-collection tube and consequently, enhance the generation of the 75 kDa derivative.

Interestingly, the reactivity of the 75 kDa region was significantly enhanced by Hep-III treatment. Previous studies have suggested that the HS content of the SDC1 ectodomain may greatly affect its susceptibility to sheddases [17,18]. To the best of our knowledge, no studies have clearly documented thrombin activity in human serum. However, based on our speculation that the 75 kDa derivative is a thrombin-cleavage fragment, this finding indirectly raises the possibility that serum from patients with sepsis may have some residual thrombin activity. Overall, we speculate that the digestion of HS by heparinase may increase the accessibility of the residual thrombin in the serum or the added thrombin as an anticoagulant in the blood collection tube to the SDC1 ectodomain, and consequently, may promote its cleavage *in vitro*.

To establish the clinical significance of sSDC1, it is essential to understand its characteristics as an antigen and the immunoreactivity of the Abs used in the measurement method. Thus, we assessed the effect of the GAG chains of sSDC1 molecules on their quantification. We showed that serum sSDC1 levels significantly increased with digestion of the HS moiety by Hep-III treatment. In agreement with the results of the WB analysis, this suggested that the HS moiety affected the reactivity of the ELISA Abs with their epitopes on the sSDC1 core protein. In addition, the fact that the effect of Hep-III on the quantitative values of sSDC1 was more pronounced in the pooled septic serum than in the pooled control serum strongly supported the idea that serum sSDC1 in sepsis may be HS-rich. Unlike Hep-III, O-Gly does not cleave the HS moiety from HS chains but rather cleaves whole HS chains at the O-glycosidic bond on the SDC1 ectodomain, regardless of the HS content. Taken together, our finding that the effect of O-Gly on serum sSDC1 levels was comparable between septic patients and control patients also supported our hypothesis that the HS moiety of sSDC1 may affect the reactivity of ELISA Abs.

Finally, to further clarify the differences in the properties of serum sSDC1 between septic and control patients, we performed IP analysis. High-MW sSDC1 molecules >200 kDa, which were precipitated by the anti-HS Ab, could be detected in the pooled septic serum, but not in the pooled control serum, providing compelling evidence that the systemic inflammatory response associated with sepsis may promote the shedding of the HS-rich SDC1 ectodomain from the cell surface. Notably, physiological sSDC1 may be relatively HS-poor compared with pathological sSDC1. This interpretation is supported by dot blot analysis performed as a supplemental experiment, which confirms that the weak signals observed in control serum reflect genuinely low HS content rather than limitations in Ab performance. Based on multiple lines of evidence [[12], [13], [14], [15], [16]], there is no doubt that inflammation-induced activation of sheddases underlies the mechanism of SDC1 shedding. However, our results seem inconsistent with the abovementioned findings that a decrease in HS is a prerequisite for SDC1 shedding [17,18]. Although we cannot rule out the possibility that the difference in HS content between pathological and physiological sSDC1 may be due to the difference in the cells from which they originate, our results suggested that the activation of sheddases may be evoked independently of the HS content on the core protein of the SDC1 ectodomain. Previous studies, such as those by Ramani et al. [18], proposed that loss or shortening of HS chains promotes SDC1 shedding by increasing protease accessibility. In contrast, our data indicate that serum sSDC1 in sepsis is enriched in HS. These seemingly contradictory findings may be reconciled by a two-step model. In the early phase of sepsis, inflammatory stimuli and endothelial injury may trigger the release of HS-rich SDC1 ectodomains from the cell surface. Once released into the circulation, these ectodomains may undergo further enzymatic processing by heparanase or thrombin, leading to the gradual loss of HS chains and the generation of lower-MW fragments. This model suggests that the HS content of circulating sSDC1 reflects not only the shedding mechanism at the cell surface but also subsequent modifications occurring in the bloodstream. Understanding this dynamic process may provide a more integrated view of SDC1 biology in sepsis. Subsequent studies will be required to elucidate the mechanisms behind the release of HS-rich sSDC1 in sepsis and how HS modulates sSDC1 function, including its role in inflammatory signaling, coagulation, and endothelial dysfunction.

More importantly, although the ELISA used in this study was designed to specifically detect pathological derivatives among pan-sSDC1, the following points should be noted when interpreting the data. First, the 75 kDa derivative, which may be induced by thrombin cleavage, may not be measured. Second, the sepsis-induced pathological increase in HS-rich sSDC1 may also be underestimated because the HS content of sSDC1 molecules may have a substantial effect on their quantitative values. In particular, caution should be exercised when using serum sSDC1 levels as biomarkers to evaluate the pathological status of sepsis. Indeed, previous studies reported that the HS content of sSDC1 molecules varies with pathological status [[29], [30], [31]]. Further studies will be required to more precisely evaluate the reactivity of the ELISA to various sSDC1 fragments.

Importantly, these findings have several implications for clinical applications of sSDC1 measurement. First, the high HS content of pathological sSDC1 may hinder Ab accessibility in standard ELISA assays, leading to underestimation of sSDC1 levels. Practical approaches to mitigate this issue include either pre-treating serum samples with Hep-III to remove HS chains or modifying the assay design to improve Ab access to HS-rich epitopes. Second, since only one commercial ELISA kit was used in this study, caution is required when generalizing these results. Future studies should evaluate multiple ELISA platforms and further optimize assay conditions to ensure accurate quantification of HS-rich sSDC1 molecules under different pathological conditions. Furthermore, in interpreting our findings, several limitations should be considered. First, the serum sSDC1 levels and molecular characteristics could be influenced by potential confounders, including patients’ comorbidities and the handling of blood samples, such as residual thrombin in collection tubes. Second, this study was conducted at a single center, which may limit the generalizability of our findings. Future studies with larger, multi-center cohorts will be necessary to validate these observations and to further assess the reproducibility of sSDC1 measurements under different pathological and clinical conditions.

In summary, we identified differences in the molecular characteristics of serum sSDC1 between patients with and without sepsis. HS is a characteristic feature of pathological sSDC1 in the sera of patients with sepsis, and HS content may confer heterogeneity to serum sSDC1 molecules and affect ELISA quantification values.

## CRediT authorship contribution statement

**Shogo Akahane:** Writing – original draft, Methodology, Investigation, Funding acquisition. **Riho Shimizu:** Methodology, Investigation. **Harue Suzuki:** Methodology. **Hiroto Matsuura:** Methodology. **Yoko Usami:** Resources, Methodology. **Nau Ishimine:** Resources, Methodology. **Takeshi Uehara:** Writing – review & editing, Resources, Investigation. **Kazuyoshi Yamauchi:** Writing – review & editing, Visualization, Validation, Supervision, Software, Resources, Project administration, Methodology, Investigation, Funding acquisition, Formal analysis, Data curation, Conceptualization.

## Ethical approval

The present study was approved by the Ethical Review Board of Shinshu University School of Medicine (approval number, 5762).

## Funding statement

This research was supported by a Grant-in-Aid for Scientific Research from the 10.13039/501100001691Japan Society for the Promotion of Science (JSPS KAKENHI Grant Number 24K10594 to K.Y.) and the Shinshu Public Utility Foundation for the promotion of medical science (to SA).

## Declaration of competing interest

The authors declare that they have no known competing financial interests or personal relationships that could have appeared to influence the work reported in this paper.

## Figures and Tables

**figs1:**
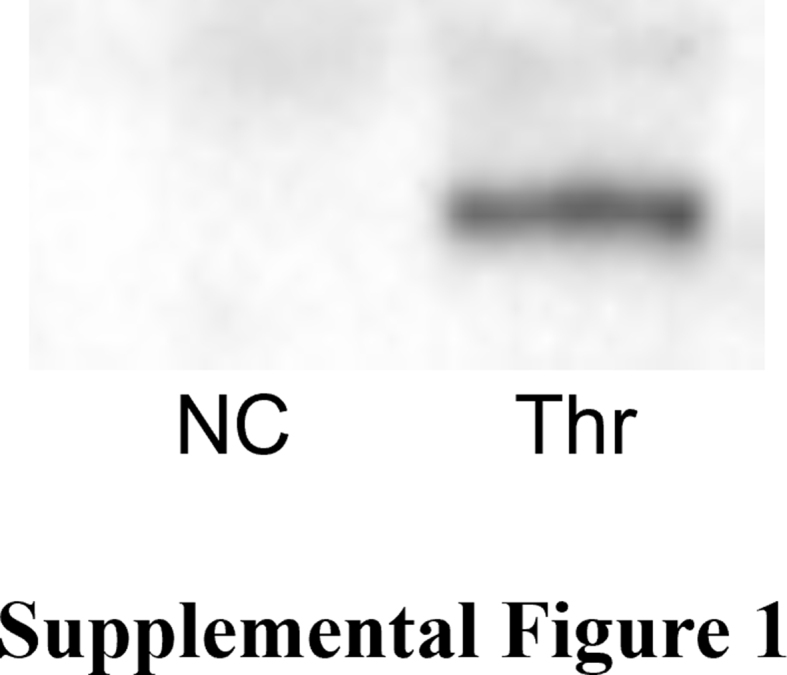


**figs2:**
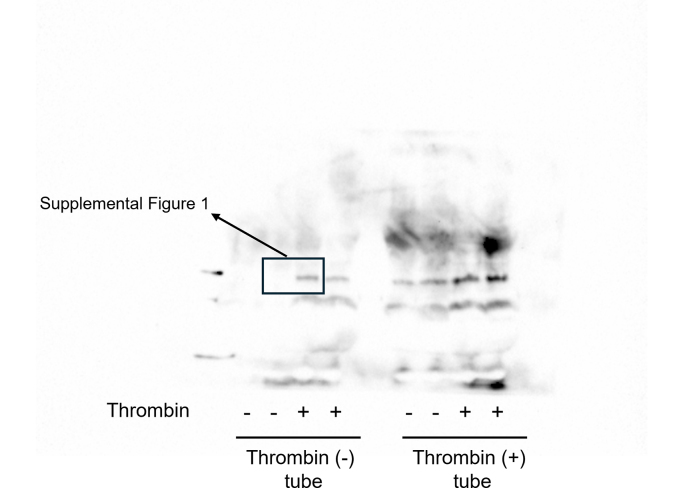


**figs3:**
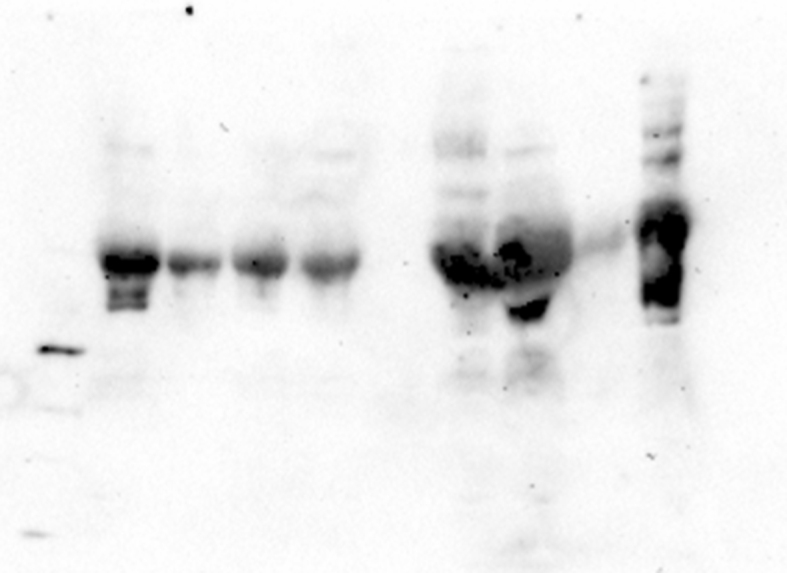


**figs4:**
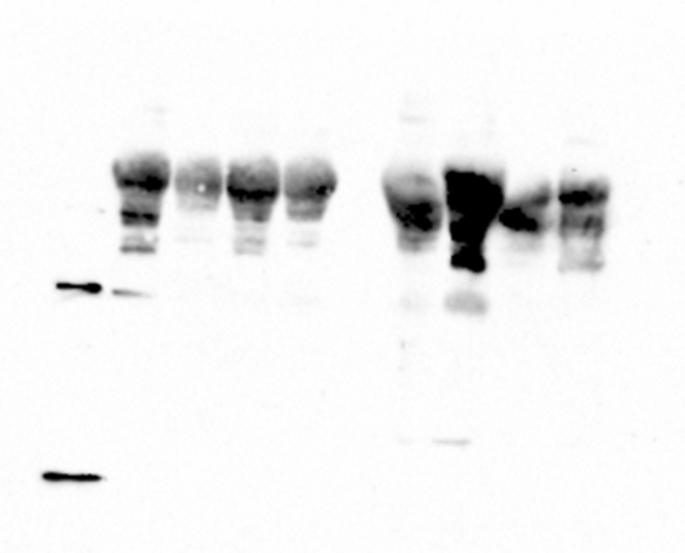


**figs5:**
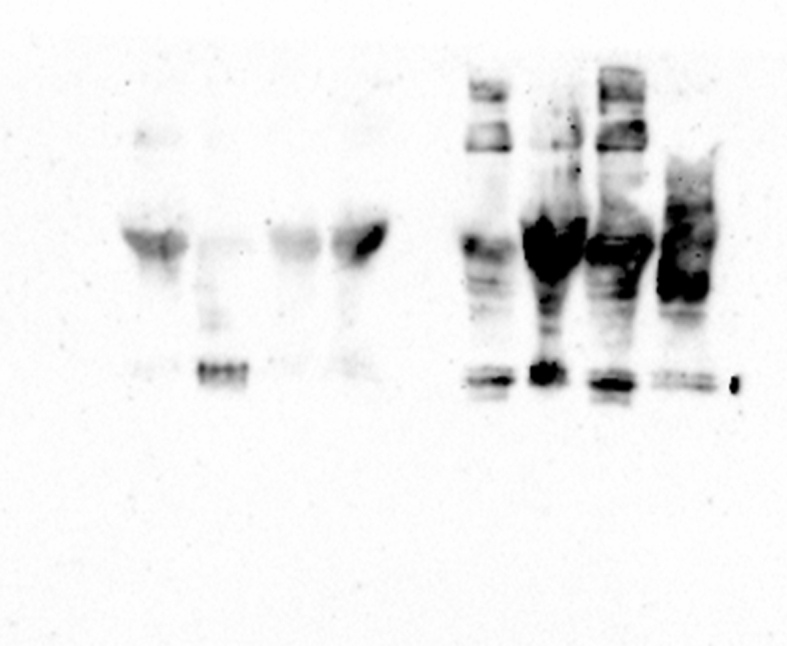


**figs6:**
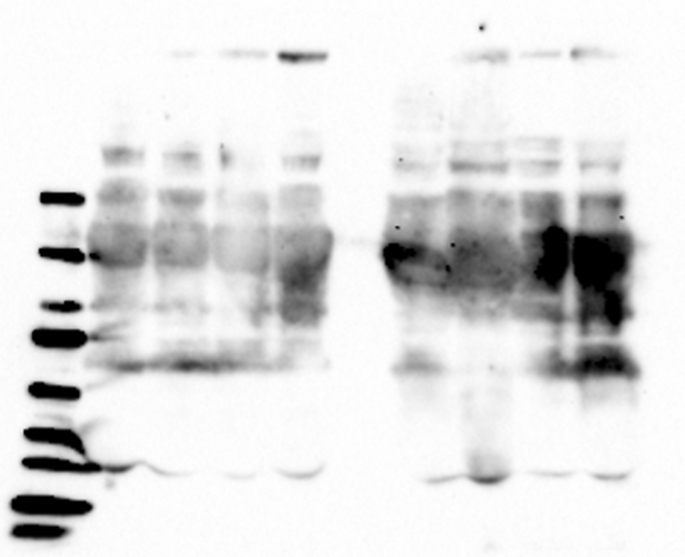


**figs7:**
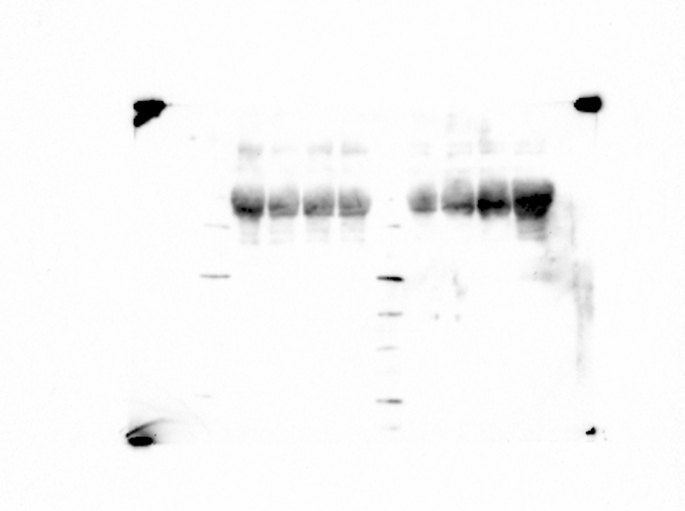


**figs8:**
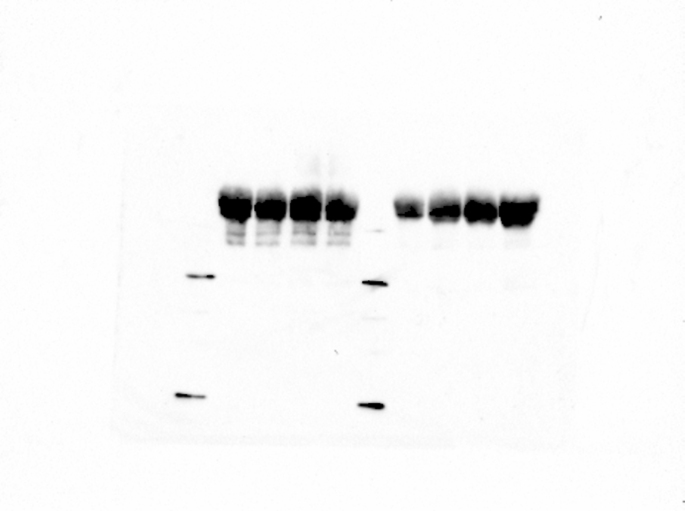


**figs9:**
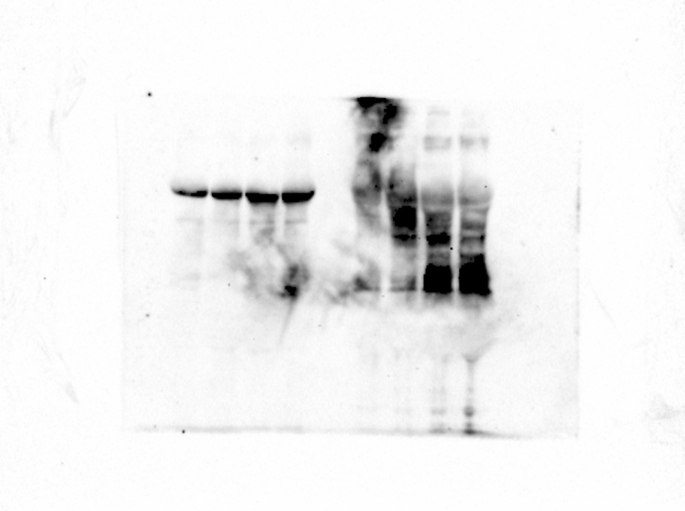


**figs10:**
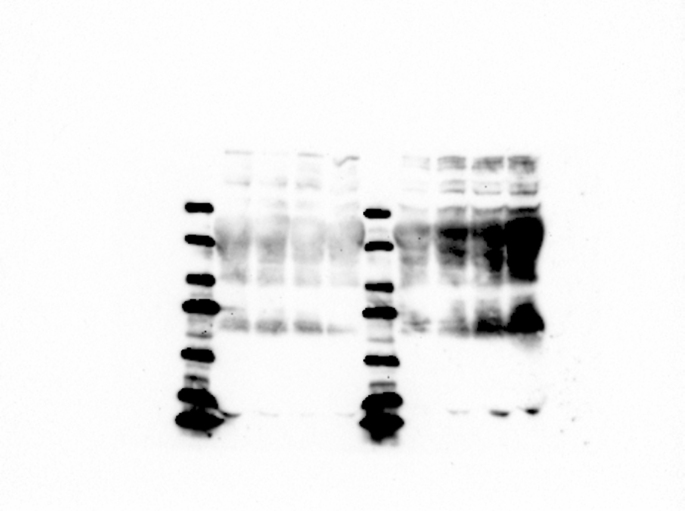


**figs11:**
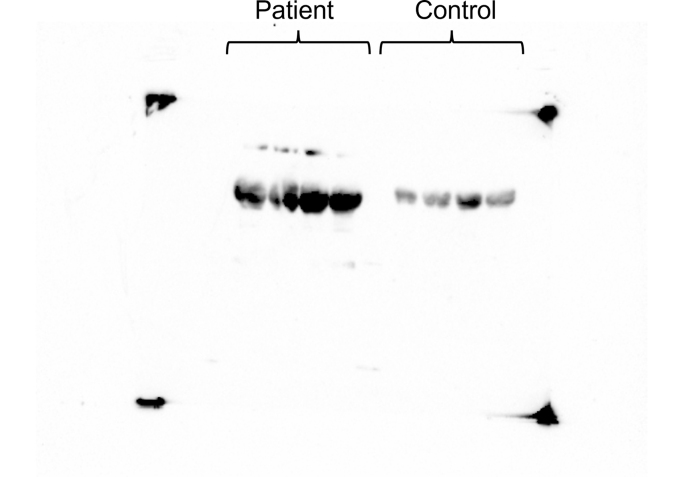


**figs12:**
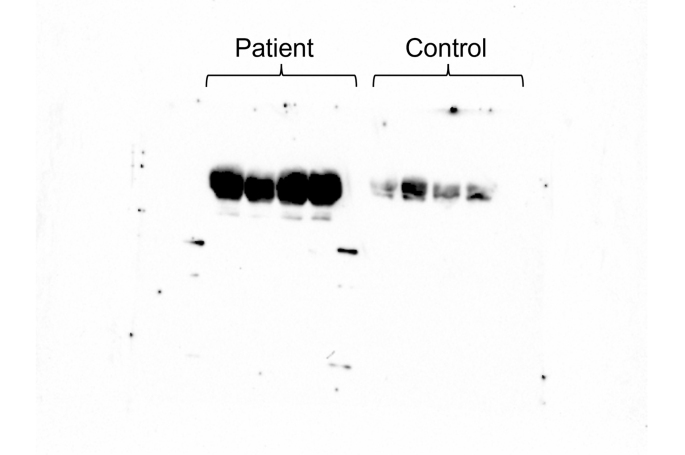


**figs13:**
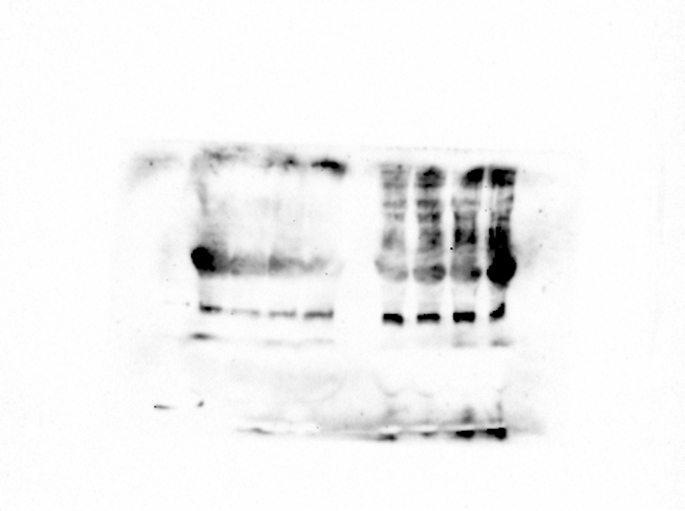


**figs14:**
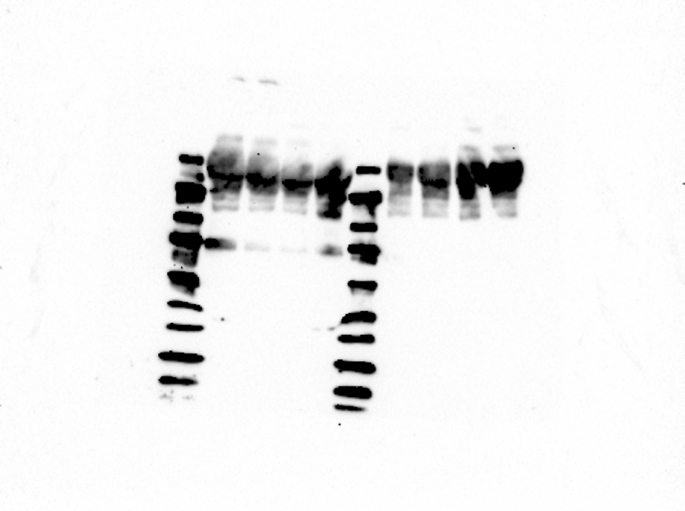


**figs15:**
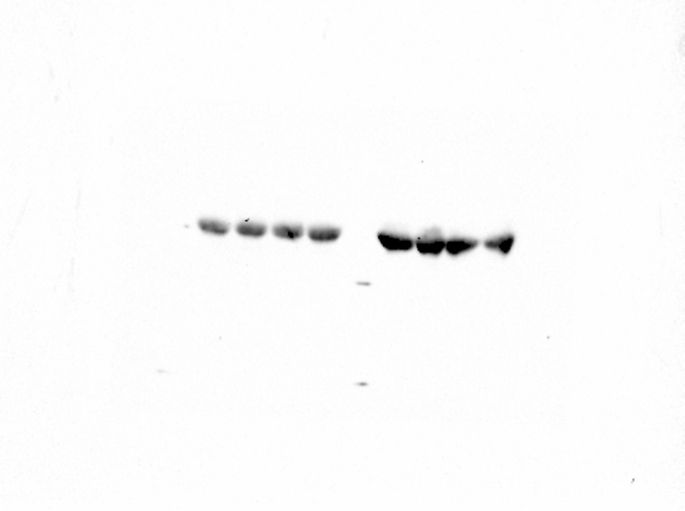


**figs16:**
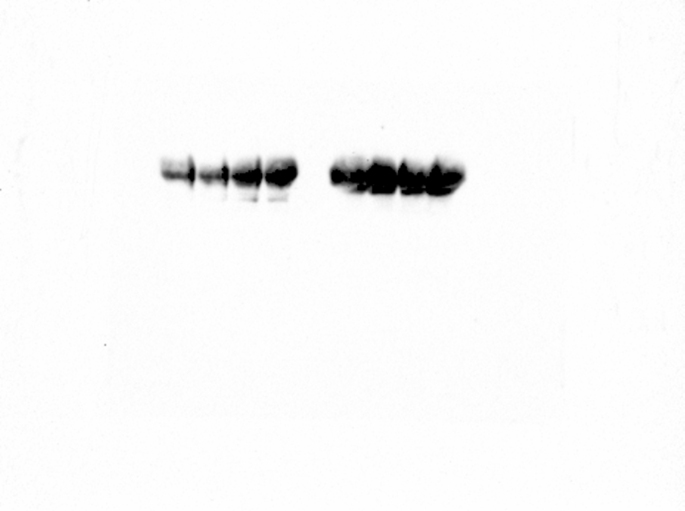


**figs17:**
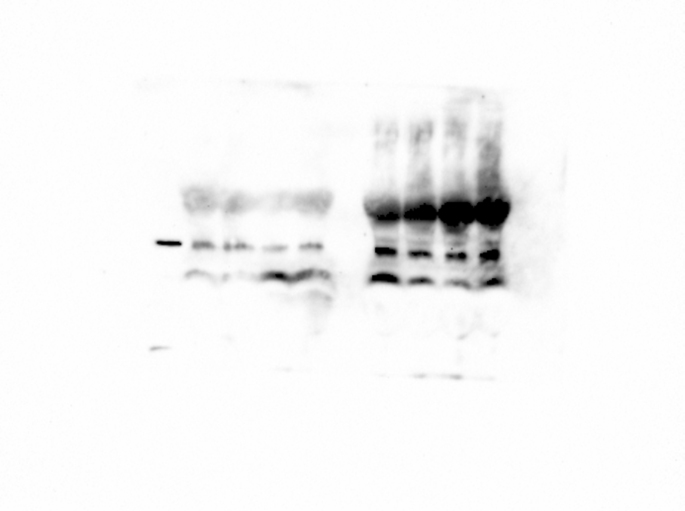


**figs18:**
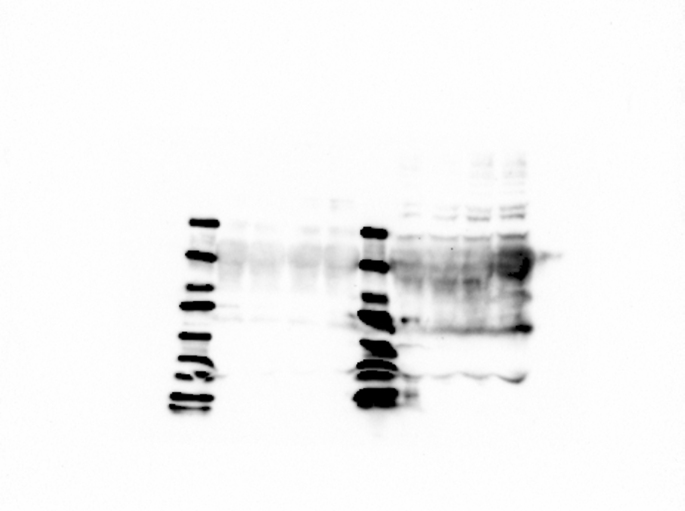


**figs19:**
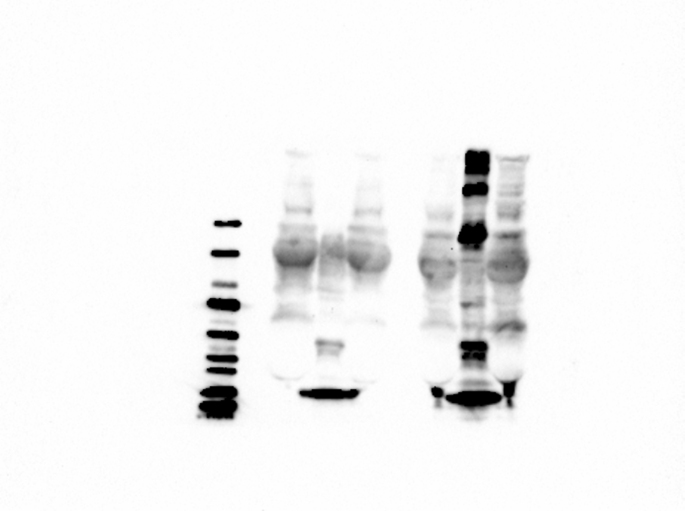


## Data Availability

Data will be made available on request.
