# Supplementary material for: Serum-soluble SDC1 in septic patients is rich in heparan sulfate, which affects the ELISA quantification values
Source: Biochem Biophys Rep. 2025 Oct 30;44:102333. doi: 10.1016/j.bbrep.2025.102333 (PMC12641194; doi:10.1016/j.bbrep.2025.102333)
Supplement: Multimedia component 1 [file mmc1.docx]

**Serum-soluble SDC1 in septic patients is rich in heparan sulfate, which affects the ELISA quantification values**

Shogo Akahane^1) #^, Riho Shimizu^1), 2)^, Harue Suzuki^1), 2)^, Hiroto Matsuura^1), 2)^, Yoko Usami^2)^,

Nau Ishimine^2)^, Takeshi Uehara^2)^, Kazuyoshi Yamauchi^1), 3) *^

^1)^ Department of Clinical Laboratory Investigation, Graduate School of Medicine, Shinshu University, Matsumoto, Japan.

^2)^ Department of Laboratory Medicine, Shinshu University Hospital, Matsumoto, Japan.

^3)^ Department of Biomedical Laboratory Sciences, School of Health Sciences, Shinshu University, Matsumoto, Japan.

***Address correspondence to**: Kazuyoshi Yamauchi, PhD, Department of Clinical Laboratory Investigation, Graduate School of Medicine, Shinshu University, 3-1-1 Asahi, Matsumoto, 390-8621, Japan, Tel: +81-263-37-2368, FAX: +81-263-37-2370

e-mail: [yamauchi@shinshu-u.ac.jp](mailto:yamauchi@shinshu-u.ac.jp)

- **Supplementary Materials and Methods**
- **Supplementary Figures**

**Supplementary Figure 1:** **Effect of thrombin addition on the 60–80 kDa derivatives of sSDC1 in plasma from healthy volunteer.**

**Supplementary Materials and Methods**

**Sample collection**

Plasma was isolated from the blood of healthy volunteers using heparin-supplemented blood collection tubes.

**Supplementary figure legend**

**Supplementary Figure 1: Effect of thrombin addition on the 60–80 kDa derivatives of sSDC1 in plasma from healthy volunteer.**

Bovine plasma thrombin (FUJIFILM Wako Pure Chemicals, Osaka, Japan) was added to the plasma samples at final concentrations of 0.1 or 1.0 U/mL, followed by incubation for 16 h at 37°C. Similarly, plasma samples were incubated with 1.0 U/mL heat-inactivated thrombin as a negative control (NC) with zero enzyme activity. The prepared samples were subjected to western blotting analysis using a polyclonal anti-SDC1 antibody.
